# *In vivo* tracing of the ascending vagal projections to the brain with manganese enhanced magnetic resonance imaging

**DOI:** 10.3389/fnins.2023.1254097

**Published:** 2023-09-15

**Authors:** Steven Oleson, Jiayue Cao, Xiaokai Wang, Zhongming Liu

**Affiliations:** ^1^Weldon School of Biomedical Engineering, Purdue University, West Lafayette, IN, United States; ^2^Department of Biomedical Engineering, University of Michigan, Ann Arbor, MI, United States; ^3^Department of Electrical Engineering Computer Science, University of Michigan, Ann Arbor, MI, United States

**Keywords:** manganese-enhanced magnetic resonance imaging, neuronal tracing, vagus nerve, vagus nerve stimulation, nucleus tractus solitarius

## Abstract

**Introduction:**

The vagus nerve, the primary neural pathway mediating brain-body interactions, plays an essential role in transmitting bodily signals to the brain. Despite its significance, our understanding of the detailed organization and functionality of vagal afferent projections remains incomplete.

**Methods:**

In this study, we utilized manganese-enhanced magnetic resonance imaging (MEMRI) as a non-invasive and *in vivo* method for tracing vagal nerve projections to the brainstem and assessing their functional dependence on cervical vagus nerve stimulation (VNS). Manganese chloride solution was injected into the nodose ganglion of rats, and T1-weighted MRI scans were performed at both 12 and 24 h after the injection.

**Results:**

Our findings reveal that vagal afferent neurons can uptake and transport manganese ions, serving as a surrogate for calcium ions, to the nucleus tractus solitarius (NTS) in the brainstem. In the absence of VNS, we observed significant contrast enhancements of around 19–24% in the NTS ipsilateral to the injection side. Application of VNS for 4 h further promoted nerve activity, leading to greater contrast enhancements of 40–43% in the NTS.

**Discussion:**

These results demonstrate the potential of MEMRI for high-resolution, activity-dependent tracing of vagal afferents, providing a valuable tool for the structural and functional assessment of the vagus nerve and its influence on brain activity.

## Introduction

The vagus nerve, a critical component of the peripheral nervous system, supports rapid communication between the brain and the body’s internal organs. It allows the brain to control physiological functions related to respiratory, cardiovascular, immune, and digestive systems (Tracey, 2002; Thayer and Lane, 2007; Chang et al., 2015; Travagli and Anselmi, 2016; Powley, 2021). It also allows these organ systems to influence the brain, shaping perception, behavior, cognition, and emotion (Craig, 2002; Critchley and Harrison, 2013; Azzalini et al., 2019; Hsueh et al., 2023). Such brain-body interactions are termed as “interoception” (Chen et al., 2021), a concept that has recently gained considerable interest in the fields of neuroscience and integrative physiology.

The vagus nerve is being increasingly recognized as a major target of bioelectric medicine (Birmingham et al., 2014). Vagus nerve stimulation (VNS), which involves delivering electrical pulses to the vagus nerve at varying levels, has been explored as a therapeutic strategy. It has been used to treat epilepsy (Morris and Mueller, 1999), depression (Rush et al., 2005), pain (Chakravarthy et al., 2015), or to promote learning and rehabilitation (Engineer et al., 2011, 2019). It has also gained attention for its potential in relieving chronic conditions affecting internal organs and systems (Premchand et al., 2014; Bonaz et al., 2016). However, the mechanism of action for VNS is incompletely understood. Optimizing the application of VNS for therapeutic effects often relies on a trial-and-error process.

The internal structure of the vagus nerve is complex, encompassing nerve fibers with diverse morphological features, fascicular organizations, and functional associations (Stakenborg et al., 2020; Havton et al., 2021). Approximately 80% of vagal nerve fibers are afferent (Berthoud and Neuhuber, 2000; Powley et al., 2019), enabling sensory neurons in the nodose ganglia (NG) to relay a variety of bodily signals to the nucleus tractus solitarius (NTS) in the brainstem (Prescott and Liberles, 2022; Ran et al., 2022). These signals, once in the NTS, can further ascend to various brain regions (Craig, 2002; Browning and Travagli, 2014; Berntson and Khalsa, 2021). The interplay between ascending (sensory) and descending (motor) pathways forms the functional neural circuits of interoception, which play a critical role in regulating both mental and physiological states (Chen et al., 2021).

Despite the crucial role of the vagus nerve in mediating brain-body interactions, current methodologies constrain our understanding of its structural and functional connectivity. Viral tracing, while useful for localizing neuronal projections (Kaelberer et al., 2018), cannot provide functional characterization. Immunohistochemistry with cFos enables the localization of neuronal responses (Cunningham et al., 2008), but lacks the capability for longitudinal measures. *In vivo* electrophysiology offers acute measures of neuronal activity (Cao et al., 2021), but is limited by its invasive nature and restricted scope. Functional magnetic resonance imaging (fMRI) offers non-invasive yet indirect measures of neural activity but lacks spatial resolution or specificity to discern fine-grained activations at brainstem nuclei (Cao et al., 2017). Diffusion MRI tractography can map structural connectivity but not functional connectivity (Zhang et al., 2020). These methodological limitations underscore the need for development and exploration of alternative methods.

Manganese-enhanced magnetic resonance imaging (MEMRI) provides an alternative for potentially addressing the limitations of the aforementioned methods (Pautler et al., 1998; Silva et al., 2004; Saar and Koretsky, 2018). Being an analog of the calcium ion (Ca^2+^), the manganese ion (Mn^2+^) can enter the calcium channels of excitable neurons, travel along axonal pathways, enter post-synaptic neurons, and continue to migrate along the circuit (Takeda et al., 1998; Pautler, 2004). In addition, the axonal transport and cellular uptake of manganese are activity dependent. Increase in neural activity results in increased transport and accumulation of manganese ions (Lin and Koretsky, 1997; Duong et al., 2000; Bearer et al., 2007). Importantly, increase in manganese ions shortens the T_1_ relaxation time, enhancing the contrast in T_1_-weighted MRI and allowing MEMRI to assess function (Silva et al., 2004). By using manganese ions as an anterograde tracer in animal studies, MEMRI following local injection of manganese has been used to trace active neural pathways in the central nervous system (Pautler et al., 1998; Watanabe et al., 2001; Saleem et al., 2002; Yu et al., 2005; Chuang et al., 2009), yielding important insight into the cytoarchitecture and function of specific brain regions and systems. Applications of MEMRI with animal models of neurological disorders have also shown axonal transport deficits in the Alzheimer’s disease (Smith et al., 2007; Wesson et al., 2010; Kim et al., 2011; Gallagher et al., 2012; Bertrand et al., 2013; Saar et al., 2015; Fontaine et al., 2017; Bearer et al., 2018), Parkinson’s disease (Pelled et al., 2007; Soria et al., 2011; Olson et al., 2016), amyotrophic lateral sclerosis (Jouroukhin et al., 2013), and more (Saar and Koretsky, 2018). However, the use of MEMRI is rarely used with humans, due to the toxicity of manganese at the dose used in animal studies.

Despite the large number of studies that have used MEMRI for the central nervous system, it has rarely been applied to the peripheral nervous system. To date, only a few studies have utilized MEMRI for neuronal tracing along the spinal cord and sciatic nerve (Matsuda et al., 2010; Cha et al., 2019; Krishnan et al., 2020). Its utility in tracing the vagus nerve remains unexplored, to the best of our knowledge, despite the critical role of the vagus in mediating brain-body interactions in health and disease. Therefore, the primary goal of the present study is to transfer the protocol of MEMRI-based neuronal tracing from the central nervous system to the peripheral nervous system, focusing on the vagus nerve and its ascending projections into the brainstem. Specifically, we aimed to demonstrate the feasibility of tracing the ascending vagal pathway via the injection of MnCl_2_ into the left or right nodose ganglion. Next, we verified the activity-dependence of this Mn^2+^ tracing and uptake by modulating vagal activity through cervical VNS. Finally, we explored the effective time window for MEMRI following the injection. The findings and methodologies presented herein may provide the methodological foundation for *in vivo* anatomical tracing and functional characterization of the ascending vagal pathway in the rat brain. Lastly, we discussed future directions for using MEMRI to investigate the neural circuits of interoception and evaluate bioelectric treatment of brain-body disorders with animal models.

## Materials and methods

### Subjects

This study involved 19 male Sprague-Dawley rats, each weighing between 320 and 400 g. All procedures received approval from our Institutional Animal Care and Use Committee. Prior to surgery, the rats were pair-housed in a single cage within an environment controlled to maintain a 12:12 h light-to-dark cycle, with the lights turned on at 6 a.m. and turned off at 6 p.m. Following surgery, each rat was housed individually.

### Experimental design

**FIGURE 1:**
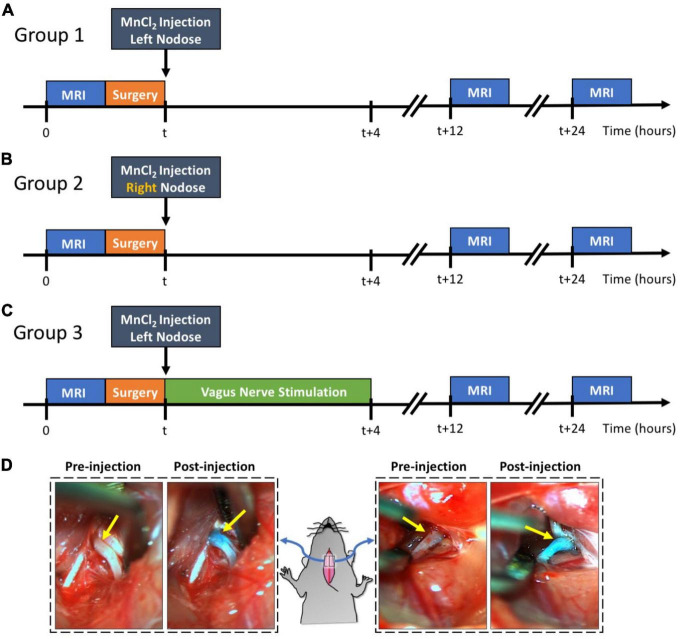
Experimental design and examples of MnCl_2_ injection into NG. Panels **(A–C)** illustrate the experimental timelines for groups 1, 2, and 3, respectively. For all three groups, the time of MnCl_2_ injection into either the left or right NG is marked as t. The time of VNS and MRI are marked relative to the time of MnCl_2_ injection. Panel **(D)** shows the right and left NG before and after MnCl_2_ injection. The NG appears as green after injection because of the green dye added to the MnCl_2_ solution.

Our study design is demonstrated in Figure 1. The rats were randomly allocated into three groups: group 1 (*n* = 9), group 2 (*n* = 5), and group 3 (*n* = 5). Groups were differentiated based on the intended goals of tracing the left vagus nerve (group 1) or the right vagus nerve (group 2) and further assessing the effect of the left vagus nerve stimulation (group 3) against a sham control (group 1). All three groups underwent identical surgical and MRI procedures.

Every rat underwent a T_1_-weighted (T_1_w) brain MRI scan prior to MnCl_2_ injection into the left or right nodose ganglion (NG). Following this pre-contrast MRI, the rats were briefly operated on to expose the NG and the cervical vagus nerve on either the left side (group 1 and 3) or right side (group 2). A solution of MnCl_2_ (1.0 μl of 500 mM, Sigma-Aldrich, St Louis, MO, USA) was injected into the exposed NG. A cuff electrode (Microprobes for Life Science, Gaithersburg, MD, USA) was then wrapped around the exposed vagus nerve. In the case of group 3, electrical stimulation was delivered via the implanted electrode to stimulate the left vagus for 4 h, after which the electrode was removed. For groups 1 and 2, the electrode was removed without administering any stimulation, thus serving as a sham control for group 3. Post-surgery, all animals were allowed to recover. At 12 and 24 h post MnCl_2_ injection, each animal underwent another T_1_w brain MRI scan. The location and level of contrast enhancement due to MnCl_2_ uptake and transport by vagal afferent nerves were assessed by evaluating the voxel-wise intensity difference between pre-contrast and post-contrast MRI.

### Anesthesia, MRI, and surgical protocol

Every rat was initially anesthetized with 5% isoflurane for 5 min prior to MRI. The isoflurane was combined with oxygen and delivered at a rate of 500 mL/min. This setting was applicable to other isoflurane dosages mentioned in this protocol unless otherwise stated. During the MRI process, anesthesia was sustained with 1–3% isoflurane. The precise isoflurane dosage varied slightly among animals and was adjusted based on monitored physiological vitals (Model-1030, SA instruments, Stony Brook, NY, USA), such as maintaining the respiratory rate around 40–60 cycles per minute and body temperature at 37–37.5°C.

For the MRI, each rat was positioned in a customized rat holder in a prone posture with its head secured using a bite bar and two ear bars. Brain MRI was conducted using a 7T horizontal-bore small animal MRI system (BioSpec 70/30; Bruker Instruments, Billerica, USA) fitted with a gradient insert (maximum gradient: 200 mT m^–1^; maximum slew rate: 640 mT^–1^ s^–1^). A ^1^H RF transmit volume coil (86 mm inner diameter) and a ^1^H RF receive-only rat-head surface coil were used for the brain MRI. The imaging session started with a localizer to identify the brainstem and other areas of interest. Then a 2D Turbo RARE pulse sequence was used to acquire T_2_-weighted (T_2_w) images covering the brainstem (TR/TE = 6637.715/32.50 ms; FA = 90°; matrix size = 192 × 192; FOV = 32 mm × 32 mm; slice thickness = 0.438 mm; slices = 64; NEX = 2; ETL = 8). A 3D RARE pulse sequence was used to acquire T_1_w images covering the same region with the same orientation as T_2_w scans (TR/TE = 300/10 ms; FA = 90°; matrix size = 192 × 192 × 64; FOV = 32 mm × 32 mm × 28 mm; NEX = 4; ETL = 8). The same MRI procedure was used both before and after injection of MnCl_2_, generating pre-contrast and post-contrast MRI images for comparison.

Immediately after the pre-contrast MRI, every animal was moved to a surgical station adjacent to the MRI room. Each rat was anesthetized using 5% isoflurane and received an injection of carprofen (10 mg/kg, IP, Zoetis, NJ, USA). The animal was then positioned supine for surgery, with anesthesia maintained at 2% isoflurane. We confirmed adequate anesthesia using a toe pinch reflex test. We then shaved the rat along its neck and cleaned the exposed skin using iodine. An incision was performed along the ventral midline, extending from the mandible to the sternum. The underlying tissue was dissected to expose the trachea and the left or right carotid artery. We identified the cervical vagus nerve next to the carotid artery and further dissected the tissue connecting the carotid artery and the cervical vagus nerve to isolate the vagus nerve. Following the vagus nerve rostrally, we identified the NG.

The NG received a 1.0 μl injection of 500 mM MnCl_2_ solution, administered using a Nanofil 10 μl sub-microliter injection system fitted with a beveled 35-gauge needle (World Precision Instruments, Sarasota, FL, USA). To visually guide and verify the injection, we mixed the MnCl_2_ solution with a green dye (fast green FCF, Sigma-Aldrich, St. Louis, MO, USA). An example of this is shown in Figure 1D. After injection, a bipolar cuff electrode (MicroProbes, Gaithersburg, MD, USA) was wrapped around the exposed vagus nerve.

For the rats in groups 1 and 2, we removed the electrode immediately after implantation and allowed the animals to recover from surgery. However, for those in group 3, we administered vagus nerve stimulation (VNS) for 4 h. We delivered electrical current pulses via the cuff electrode at an inter-pulse duration (IPD) of 50 ms, a pulse amplitude (PA) of 1 mA, a pulse width (PW) of 0.5 ms, and a frequency of 5 Hz. The stimulation pattern consisted of 20 s on and 40 s off. After VNS, we removed the electrode, and the animals were allowed to recover. Note that groups 1 and 2 did not receive stimulation but underwent the same surgical procedure and electrode implantation as those in group 3. This made groups 1 and 2 comparable, with group 1 serving as a sham control for group 3.

### Image processing and statistical analysis

For individual animals, we processed MRI data using a combination of FSL (Smith et al., 2004), AFNI (Cox, 1996), and MATLAB scripts developed in-house. The T_2_-weighted (T_2_w) images were linearly registered to a rat brain template (Valdes-Hernandez et al., 2011) using FLIRT. Furthermore, we registered the T_1_w images to the T_2_w images from the same rat using FLIRT. After this, we registered them to the brain template based on the linear transformation determined from the T_2_w images to the template. After preprocessing, we normalized the intensity of each T_1_w slice by its average voxel intensity within the same slice.

The voxel-wise intensity increase in the post-contrast T_1_w MRI was calculated relative to the pre-contrast T_1_w MRI using the following Equation,

$$\Delta \,I\,\left(r\right)=\frac{I_{p\,o\,s\,t}\,\left(r\right)-I_{p\,r\,e}\,\left(r\right)}{I_{p\,r\,e}\,\left(r\right)}\times 100\%.$$

Here, *I*_*post*_ and *I*_*pre*_ represent the voxel intensity at each location *r* in the post- and pre-contrast MRI, respectively. We calculated the relative enhancement Δ*I*(*r*) for each voxel in each animal and then averaged this across animals in each group.

For each group, we performed a right-tailed paired *t*-test for comparing the differences between *I*_*post*_ and *I*_*pre*_ for every voxel at a 98% confidence level (or a significance level of alpha = 0.02). The statistically significant voxels were color-coded and shown on the rat brain template to highlight the brain regions where Mn^2+^ ions were accumulated.

We also compared the level of enhancement between groups. First, we identified the highest contrast enhancement (in terms of Δ*I*) within a region of interest (primarily the NTS) for each group. We then compared these results between the different groups using a right-tailed two-sample *t*-test with a confidence level of 95% (alpha = 0.05).

## Results

**FIGURE 2:**
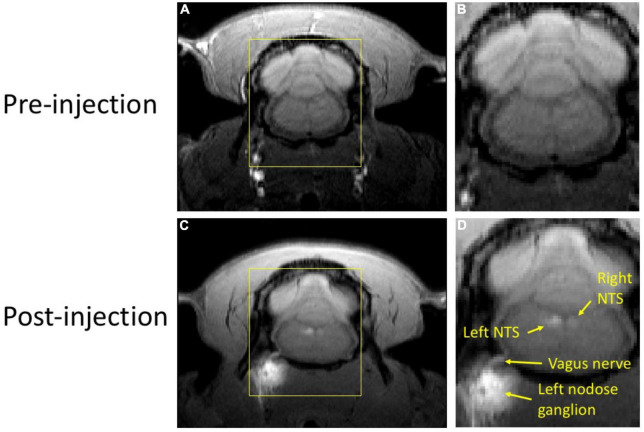
T1w MRI before and after MnCl_2_ injection into the left NG in a single animal. Panels **(A,C)** show a single slice of T1w MRI covering the brainstem and NG before and (24 h) after injection, respectively. Panels **(B,D)** zoom in the area within the yellow bounding box. In panel **(D)**, locations of visible enhancement are highlighted with arrows and annotated with anatomical labels.

In our study with rats, we evaluated the potential of MEMRI for tracing vagal afferent projections within the brain. Since vagal afferent neurons are located within the NG, we injected MnCl_2_ into this region and used MRI to identify and measure T_1_w contrast enhancement, which results from neuronal uptake and axonal transport of Mn^2+^ ions along the ascending vagal pathways. Figure 2 provides a representative example from a single rat that received a MnCl_2_ injection into its left NG. At 24 h following the injection, T_1_w MRI showed notable contrast enhancement at the left NG, the left cervical vagus nerve, and the left NTS, as well as at the right NTS, relative to the pre-injection baseline. The enhancement at the ipsilateral NTS was more substantial than that at the contralateral NTS. These results suggest that the Mn^2+^ ions were taken up by sensory neurons in the left NG, transported along the ascending vagal nerves, and then taken up by post-synaptic neurons in the left NTS, which received direct vagal projections. Further uptake by neurons in the right NTS might occur through its neuronal connection with the left NTS, or through weak connections with the left NG.

**FIGURE 3:**
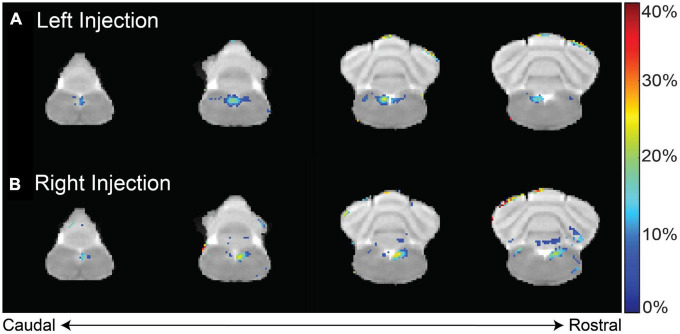
T1w contrast enhancement after MnCl_2_ injection into the left or right NG. Panels **(A,B)** show the group-averaged percentage of T1w enhancement at 12 h after MnCl_2_ injection into the left (group 1) or right (group 2) NG relative to the pre-injection baseline, respectively. Highlighted in color are voxels of statistically significant enhancement (paired *t*-test, right-tailed *p* < 0.02), where the color indicates the percentage of contrast enhancement. The four slices cover the lower brainstem and cerebellum.

This finding was corroborated in two animal groups that received MnCl_2_ injections either in the left (*n* = 9) or the right (*n* = 5) NG (Figure 3). When compared to the pre-injection baseline, the NTS and the area postrema (AP) exhibited significant enhancement in T_1_w MRI (paired *t*-test, *p* < 0.02). At the 12 h mark following the MnCl_2_ injection, significant contrast enhancement was evident at the ipsilateral NTS and to a lesser degree at the contralateral NTS. At the group level, the strongest contrast enhancement was observed at the ipsilateral NTS, with enhancement levels of 24.75 ± 4.18% or 27.06 ± 1.28% following left or right NG injection, respectively. These findings demonstrate the high spatial specificity of MEMRI in localizing nuclei that receive vagal afferent projections.

**FIGURE 4:**
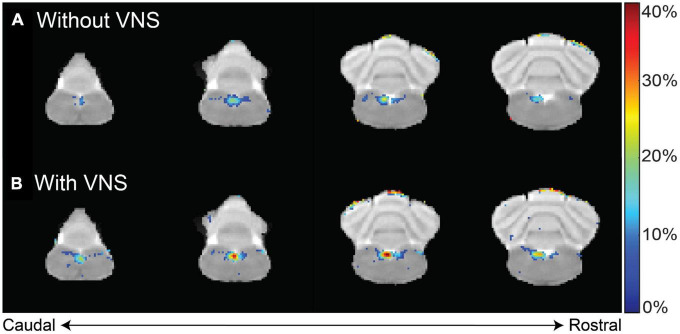
Difference in contrast enhancement with vs. without VNS. Panels **(A,B)** summarize the group-averaged contrast enhancement at 12 h after MnCl_2_ injection into the left NG without or with 4 h of VNS applied to the left cervical vagus, respectively. Highlighted in color are voxels of statistical significance (paired *t*-test, right-tailed *p* < 0.02), where the color indicates the percentage of enhancement relative to the pre-injection baseline.

**FIGURE 5:**
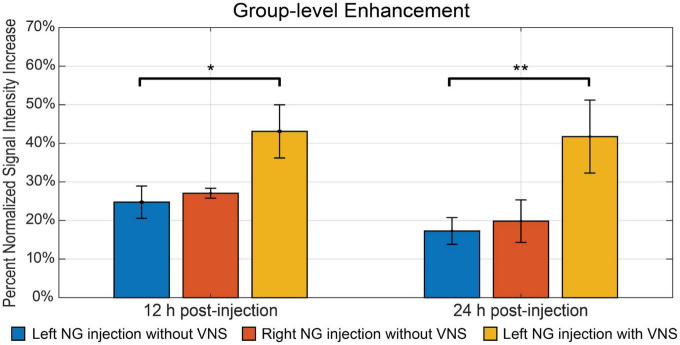
The summary of intensity increase in NTS on the ipsilateral side of nodose ganglion injection. The bar plot shows the mean and standard error of the mean of maximum intensity increase within NTS. The comparison includes all three groups. The blue bar indicates group 1, where rats received left nodose ganglion injection but not VNS. The red bar indicates group 2, where rats received right nodose ganglion injection but not VNS. The yellow bar indicates group 3, where rats received left nodose ganglion injection and VNS on the same side. The bar plots contain comparisons at both 12 h and 24 h post-injection. *Denotes that the difference is statistically significant on the right-tailed two-sample *t*-test (*p* < 0.05), **denotes that the difference is statistically significant on the right-tailed two-sample *t*-test (*p* < 0.01).

Furthermore, we investigated whether the observed Mn^2+^ uptake was dependent on activity, by applying electrical current pulses (IPD = 50 ms; PW = 0.5 ms; PA = 1 mA; 5 Hz; alternating 20 s ON and 40 s OFF over a 4 h period) to the left cervical vagus nerve (group 3, *n* = 5). We then compared the resulting contrast enhancement to the sham group that did not receive stimulation (group 1, *n* = 9). At 12 h following the MnCl_2_ injection, the left NTS exhibited the greatest contrast enhancement, with 43.10 ± 6.89% in the presence of VNS relative to 24.75 ± 4.18% without VNS (Figure 4). A similar trend was observed at 24 h after the MnCl_2_ injection, where contrast enhancement at the left NTS was higher with VNS (41.74 ± 9.46%) than without VNS (17.28 ± 3.47%). The effect of VNS was significant at both 12 h (*p* = 0.0160) and 24 h (*p* = 0.0061) post-MnCl_2_ injection (Figure 5). These findings suggest that the Mn^2 +^ uptake at the NTS was sensitive to functional changes in vagal nerve activity as a result of VNS.

Finally, we compared the contrast enhancement at 12 h and 24 h post-MnCl_2_ injection to assess the time-dependent uptake, transport, and accumulation of Mn^2+^. Figure 5 depicts the contrast enhancement at the ipsilateral NTS across all three groups. In the absence of VNS, spontaneous vagal activity resulted in slightly higher contrast enhancement at 12 h post-injection, compared to the 24 h mark. The effect of VNS was more pronounced at 24 h, resulting in an additional 24.46% enhancement, compared to 18.35% at 12 h (Figure 5). This suggests that, following unilateral injection of MnCl_2_ into the NG, Mn^2+^ ions are absorbed by post-synaptic neurons in the NTS within a 12-h period, and that MEMRI is highly sensitive to the cumulative effect of vagal afferent activity over a 24-h period.

## Discussion

This study extends a large number of studies that have used MEMRI for neuronal tracing in the central nervous system (Pautler et al., 1998; Silva et al., 2004; Saar and Koretsky, 2018) and demonstrates the feasibility for a new application of mapping vagal afferent projections in rats. By injecting MnCl_2_ into the NG and applying subsequent MRI imaging, we were able to visualize and quantify the T_1_w contrast enhancement, which signifies the neuronal uptake and axonal transport of Mn^2+^ ions along the vagal nerve pathways. Our findings confirm the robustness and spatial specificity of this technique, showing significant contrast enhancement at the NTS and AP which receive direct vagal projections. Importantly, we also discovered that Mn^2+^ uptake measured by MRI was sensitive to variations in vagal nerve activity induced by vagus nerve stimulation, demonstrating the feasibility of using MEMRI to not only trace neuronal pathways but also monitor changes in neural activity along the pathways. Although prior studies have demonstrated the utility of MEMRI for tracing spinal and sciatic nerves (Matsuda et al., 2010; Cha et al., 2019; Krishnan et al., 2020), our study offers the first demonstration for tracing the vagus nerve.

MEMRI provides several advantages for tracing the vagus nerve. Foremost, MEMRI uses MRI and thus is non-invasive. The injection of Mn^2+^ ions into the nodose ganglion, a peripheral structure housing the cell bodies of vagal afferent neurons, involves a relatively minor surgical intervention without major complications. Post-injection, the animal typically recovers well ahead of the subsequent MRI scans, providing an ample time window (1–2 days) for introducing various bodily states or applying various settings of neuromodulation. The impacts of these states or interventions on vagal nerve transport and activity can be assessed through MEMRI. Moreover, MEMRI can be conducted over multiple sessions, enabling chronic experiments and repeated monitoring of longitudinal changes in neural activity and anatomical connections.

MEMRI presents an opportunity for poly-synaptic neuronal tracing in an activity-dependent manner, as previous studies tracing CNS pathways have demonstrated (Pautler et al., 1998; Watanabe et al., 2001; Saleem et al., 2002; Yu et al., 2005; Chuang et al., 2009). In our investigation, we found evidence of Mn^2+^ induced contrast enhancement not only in the ipsilateral NTS, which receives direct vagal projections from the Mn^2+^ injected NG, but also in the contralateral NTS. This observation implies that *trans-*synaptic uptake of Mn^2+^ occurs by neurons in the ipsilateral NTS. Extensive inter-connections between the left and right NTS further facilitate Mn^2+^ transportation to the opposing side. It is also likely that the left (or right) nodose ganglion projects to the right (or left) NTS; nevertheless, the contralateral projections are much less common than the ipsilateral projections (Altschuler et al., 1989). Also supporting poly-synaptic tracing, we observed contrast enhancement beyond the NTS and AP regions, reaching into other focal areas within the brainstem or cerebellum (data are not shown). However, these additional enhancements lacked consistency across animals and did not achieve statistical significance.

While our current study was unable to consistently trace vagal projections beyond the lower brainstem, we believe that future studies may overcome this limitation through refinement of the contrast agent or the administration method of manganese. A potential strategy could involve the slow release of Mn^2+^ ions, extending the period during which neurons can uptake and transport the manganese injected into the nodose ganglion. Alternative carriers for Mn^2+^ ions such as manganese dipyridoxyl diphosphate (MnDPDP) (Olsen et al., 2008; Pochwat et al., 2015) or Mn^2+^ encapsulated in nanogels (Eguchi et al., 2019) could be explored. Additionally, the use of osmotic pumps could facilitate slow and controlled delivery of Mn^2+^, potentially enhancing the efficacy of tracing (Vousden et al., 2018). Such alternatives may also help lower the dose or toxicity of Mn^2+^ to enable broader and safer applications in preclinical studies.

Given the central role of the vagus in interoception (Chen et al., 2021), MEMRI may provide a valuable tool for investigating the functional neural circuits that allow the brain to interact with visceral organs for both physical and mental health. The NTS, which receives vagal projections, maps visceral sensations by organs (Ran et al., 2022). Physiological or pathophysiological states concerning the brain’s interaction with multiple organs may manifest a signature in the vagal relay onto the NTS, which may be captured by MEMRI with local injection to the nodose ganglion. Since the vagal afferent neurons branch out to innervate different organs, the axonal transport from the nodose ganglion to the NTS cannot be attributed to a single organ. However, it is possible to perturb a specific organ and pinpoint the resulting changes in Mn^2 +^ transport and accumulation to the organ under perturbation. One of such perturbation is to stimulate the different branches of the vagus. Taking the gut as an example, we may apply subdiaphragmatic VNS to perturb the gut, without directly affecting the cardiovascular or respiratory systems, and assess the resulting effects using MEMRI. To be even more specific, one may also stimulate the nerve endings of the vagal afferent innervation on the gut and use MEMRI to evaluate the ascending vagal activity (Cao et al., 2017, 2021; Powley, 2021). Moreover, the vagus is not the only neural pathway for brain-body interactions. Using a similar protocol as in this study, we anticipate that local injection of MnCl_2_ into the dorsal root ganglion may be used to trace and assess the ascending spinal pathway, which awaits exploration and confirmation by future studies.

## Data availability statement

The raw data supporting the conclusions of this article will be made available by the authors, without undue reservation.

## Ethics statement

The animal study was approved by the Purdue University Animal Care and Use. The study was conducted in accordance with the local legislation and institutional requirements.

## Author contributions

SO: Methodology, Writing—original draft, Data curation, Formal Analysis, Investigation, Visualization. JC: Conceptualization, Data curation, Investigation, Methodology, Writing—review and editing. XW: Visualization, Validation, Writing—review and editing. ZL: Writing—review and editing, Conceptualization, Funding acquisition, Methodology, Project administration, Resources, Supervision, Writing—original draft.
